# A novel neurotherapeutic for multiple sclerosis, ischemic injury, methamphetamine addiction, and traumatic brain injury

**DOI:** 10.1186/s12974-018-1393-0

**Published:** 2019-01-23

**Authors:** Arthur A. Vandenbark, Roberto Meza-Romero, Gil Benedek, Halina Offner

**Affiliations:** 1 Neuroimmunology Research, R&D-31, VA Portland Health Care System, 3710 SW U.S. Veterans Hospital Rd., Portland, OR 97239 USA; 2 Department of Neurology, Oregon Health & Science University, 3181 SW Sam Jackson Park Rd, Portland, OR 97239 USA; 3 Department of Molecular Microbiology & Immunology, Oregon Health & Science University, 3181 SW Sam Jackson Park Rd, Portland, OR 97239 USA; 4 Present Address: Tissue Typing and Immunogenetics Laboratory, Hadassah Medical Center, Jerusalem, Israel; 5 Department of Anesthesiology and Perioperative Medicine, Oregon Health & Science University, 3181 SW Sam Jackson Park Rd, Portland, OR 97239 USA

**Keywords:** Multiple sclerosis, Ischemic injury, Methamphetamine addiction, Traumatic brain injury, Inflammation, Macrophage migration inhibitory factor, Neurotherapy, Partial MHC class II constructs, DRhQ

## Abstract

Neurovascular, autoimmune, and traumatic injuries of the central nervous system (CNS) all have in common an initial acute inflammatory response mediated by influx across the blood-brain barrier of activated mononuclear cells followed by chronic and often progressive disability. Although some anti-inflammatory therapies can reduce cellular infiltration into the initial lesions, there are essentially no effective treatments for the progressive phase. We here review the successful treatment of animal models for four separate neuroinflammatory and neurodegenerative CNS conditions using a single partial MHC class II construct called DRa1-hMOG-35-55 or its newest iteration, DRa1(L50Q)-hMOG-35-55 (DRhQ) that can be administered without a need for class II tissue type matching due to the conserved DRα1 moiety of the drug. These constructs antagonize the cognate TCR and bind with high affinity to their cell-bound CD74 receptor on macrophages and dendritic cells, thereby competitively inhibiting downstream signaling and pro-inflammatory effects of macrophage migration inhibitory factor (MIF) and its homolog, d-dopachrome tautomerase (D-DT=MIF-2) that bind to identical residues of CD74 leading to progressive disease. These effects suggest the existence of a common pathogenic mechanism involving a chemokine-driven influx of activated monocytes into the CNS tissue that can be reversed by parenteral injection of the DRa1-MOG-35-55 constructs that also induce anti-inflammatory macrophages and microglia within the CNS. Due to their ability to block this common pathway, these novel drugs appear to be prime candidates for therapy of a wide range of neuroinflammatory and neurodegenerative CNS conditions.

## Introduction

Trauma, vascular insufficiency, drug abuse, and autoimmune diseases that cause damage to the central nervous system (CNS) may have catastrophic consequences. Unfortunately, there are few effective treatments available that can ameliorate these conditions once the initial damage has occurred. In this review, we introduce a novel neurotherapeutic approach that has shown efficacy for treatment in animal models of acute and chronic multiple sclerosis (MS), ischemic injury including transient and permanent stroke, methamphetamine addiction, and traumatic brain injuries by modulating a key neuroinflammatory pathway shared by all of these conditions.

### Validation of RTL concept in various rodent models for multiple sclerosis

Starting in the mid-1990s, our laboratory designed and expressed an extensive set of unique soluble, tunable recombinant protein constructs that mimic the major histocompatibility complex (MHC) class II/peptide interface with a cognate T cell receptor [1–3]. We designated these constructs as recombinant T cell receptor ligands (RTLs). The original goal was to regulate the activation and function of selected pathogenic T cells in an MHC-restricted, antigen-specific manner. To start, we chose the experimental autoimmune encephalomyelitis (EAE) model for which there were a number of different combinations of encephalitogenic peptides and their corresponding MHC class II restriction molecules. Our first construct, RTL201, comprised of the covalently linked RT-1B β1 and α1 domains from the Lewis rat strain covalently linked to the dominant encephalitogenic guinea pig myelin basic protein (MBP) 69-89 peptide, was found to inhibit activation and proliferation of MBP-69-89-specific T cells and prevent and reverse EAE induction [4, 5]. These studies revealed several key points regarding RTL therapy. First, the combination of both MHC and peptide components of the RTL were required for successful treatment of EAE. Second, RTL treatment of EAE drastically decreased the number of encephalitogenic cells that migrated from the periphery into the CNS. Third, RTL therapy decreased both proliferation and pro-inflammatory cytokine responses of cultured MBP-specific lymph node T cells, strongly suggesting direct modulation of encephalitogenic T cells. Finally, we demonstrated direct binding of RTL201 to a soluble single-chain T cell receptor (TCR) from an encephalitogenic MBP-69-89-specific T cell clone using surface plasmon resonance, thereby proving selective RTL targeting of its cognate TCR [6].

This first success of the concept led to many more constructs for treatment of EAE as well as other inflammatory conditions. For SJL/J mice, we produced I-A^s^ β1 and α1 domains linked to proteolipid protein (PLP)-139-151 (RTL401), PLP-178-191 (RTL402), or myelin basic protein (MBP)-84-104 peptide (RTL403) that could treat relapsing EAE induced by whole spinal cord homogenates as well as by the respective encephalitogenic peptides [7, 8]. Moreover, we found that SJL/J mice immunized with multiple encephalitogenic peptides could be treated with single RTLs targeting any of the contributing pathogenic T cells, indicating a bystander suppression effect that was eventually determined to be mediated through a cytokine switch involving upregulation and secretion of IL-10 and IL-13 [7–9]. Finally, RTL401 was found to be highly effective in promoting repair of myelin and axonal damage in chronic EAE [10].

For C57BL/6 mice, we designed RTL551 (I-A^b^ β1 and α1 domains linked to mouse (m)MOG-35-55 peptide), which were highly effective for treating acute and chronic EAE induced by active immunization with mouse (m)MOG-35-55 peptide or passive EAE induced after transfer of MOG-specific T cell lines [11]. We thus demonstrated that RTL551 treatment could selectively reduce secretion of IL-17 and tumor necrosis factor (TNF)α and expression of many chemokines and chemokine receptors by transferred green fluorescent protein (GFP)-labeled encephalitogenic T cells, resulting in a dissolution of existing cellular lesions in spinal cords. Of key importance, RTL551 downregulated expression of VCAM-1 and ICAM-1 by vascular endothelial cells of the blood-brain barrier. These studies demonstrated the importance of RTL therapy for blocking migration of encephalitogenic T cells into the CNS and removal of existing inflammatory cells within spinal cord lesions.

Partial (p)MHC constructs containing DRβ1*1501 and DRα1 domains linked to human (h) or (m)mouse MOG-35-55 peptides or to MBP-85-99 peptide were also designed for studies using human T cells [12, 13] and for treatment of EAE in humanized transgenic DR2 *1501 and *1502 class II expressing mice [14, 15]. In each set of studies, these various MHC-matched RTL constructs were found to prevent or reverse clinical and histological severity of EAE mediated by the targeted T cell specificity [15–17]. In contrast, no modulation of EAE was observed using control RTLs comprised of a matched or mismatched MHC class II β1/α1 moiety linked to non-cognate encephalitogenic peptides or to non-encephalitogenic peptides, thus confirming selective T cell targeting.

### RTL signaling through the T cell receptor

To evaluate RTL signaling through the TCR, we used RTL201 to activate the A1 T cell hybridoma specific for MBP-72-89. Incubation with RTL induced a partial activation that included a CD3*ζ* p23/p21 ratio shift, ZAP-70 phosphorylation, internal calcium mobilization, NFAT activation, and transient IL-2 production [18]. In comparison, incubation of the A1 hybridoma cells with α-CD3ε produced full activation of these cellular events, with pronounced external and internal calcium mobilization, activation of NFκB and extracellular-regulated kinase (ERK), as well as long-term increased IL-2 production. These results demonstrate that RTLs can induce signaling directly through the TCR to deplete intracellular calcium stores without fully activating the T cells. The resulting Ag-specific partial activation of the transcription factor NFAT uncoupled from the activation of NFκB or ERKs constitutes a unique downstream activation pattern that can account for the inhibitory RTL effects on encephalitogenic CD4^+^ T cells.

These findings were corroborated and extended using RTL303 (DR2 β1α1-MBP-72-89) to inhibit activation of an MBP-85-99-specific, DR2-restricted human T cell clone [5]. Incubation with RTL again induced a partial activation characterized by rapid TCRζ-chain phosphorylation, calcium mobilization, and reduced extracellular signal-related kinase activity, as well as IL-10 production, but did not induce proliferation, Th1 cytokine response, or IL-2Rα expression. Upon restimulation with antigen-presenting cells (APC) primed with the MBP-85-99 peptide, the RTL-pretreated Th1 clones had reduced proliferation and IFN-γ secretion, but continued IL-10 secretion and a normal expression level of IL-2Rα. Antigen-specific IL-10 secretion by Th1 clones in response to RTL treatment confirms a “cytokine shift” to IL-10 that can account for “bystander suppression” as was seen in rodents that continued even after restimulation of the RTL-pretreated clones with APC/Ag.

### Clinical trial with RTL1000

In 2007–2009, we carried out a phase 1 double-blind, placebo-controlled, single-ascending dose clinical trial that involved 34 relapsing-remitting and secondary progressive male and female MS individuals, each receiving RTL1000 (DR2 β1α1-hMOG-35-55) or vehicle [19, 20]. The trial design involved 6 cohorts (randomized 4:2 to receive i.v. doses of 2, 6, 20, 60, 100, or 200 mg drug vs. vehicle). The primary objective was to evaluate the safety profile of RTL1000, and the secondary objectives were to evaluate its PK profile and immunological parameters in a subset of participants. The results of this trial established that RTL1000 was safe (no exacerbations or increased MRI lesions or severe adverse events; no significant induction of anti-drug antibodies) and well tolerated at a dose of ≤ 60 mg, with a half-life of < 10 min.

### Discovery of the RTL receptor, CD74, and the involvement of MIF

We determined that RTLs bind predominantly to the cell surface of monocytes, dendritic cells, and B cells in vitro [21] in a saturable manner, thus accounting for rapid partitioning from plasma to the cellular compartment (half-life < 10 min) [20]. Moreover, RTL binding to mouse APCs inhibited T cell activation and passive transfer of clinical and histological signs of EAE [21]. Taken together, these findings suggested a cell-associated RTL receptor which initiates peptide-dependent T cell tolerance involving cell-cell interactions beyond simple ligation of the TCR by soluble RTLs. RTL receptors therefore may be important for maintaining homeostasis and inducing T cell tolerance.

Further studies revealed that RTLs bind tightly to a molecular complex comprised of CD74, surface histones, and MHC class II itself [17]. This complex was expressed predominantly on CD11b^+^ monocytes and was required for treatment of EAE with a mouse version of RTL1000. RTL constructs with or without tethered antigenic peptide rapidly downregulated CD74 in a dose-dependent hierarchical manner, and blocked signaling of the inflammatory cytokine, macrophage migration inhibitory factor (MIF), and its homolog, d-dopachrome tautomerase (D-DT), for which CD74 serves as the cognate receptor. Furthermore, a significant structure activity relationship (SAR) was established between RTL-modulated CD74 levels on CD11b^+^ CNS cells and clinical severity of EAE. These results demonstrate that RTL constructs trigger both peptide-dependent and peptide-independent regulatory pathways that contribute to T cell tolerance and EAE treatment effects.

### Discovery and development of DRa1 constructs

With the focus on CD74 as the major receptor for two-domain partial (p)MHC class II constructs, we discovered that only the α1 domain but not the β1 domain could bind to immune-precipitated CD74 [17]. This led to the demonstration that the MHC class II DRα1 domain alone retains the inhibitory activity of the pMHC constructs for MIF and D-DT binding and signaling [22]. These activities of the recombinant DRα1 domain could be destroyed by trypsin digestion but were enhanced nearly 50-fold by covalently linking the MOG-35-55 peptide to the N-terminus of DRα1. This new construct exhibited discrete secondary α-helical and β-sheet structure as assessed by circular dichroism that was similar to secondary structure observed in the two domain pMHC. These data suggested a conformational determinant on DRα1-MOG-35-55 that is responsible for optimal binding to CD74 and antagonism of MIF effects, resulting in reversal of clinical and histological signs of EAE. Moreover, because the DRα1 domain as well as the MOG-35-55 moiety are present and invariable in all humans, this construct should not be recognized as foreign and treatment using this DRα1 construct would not require HLA matching in potential recipients. This feature of DRα1-MOG-35-55 construct is a substantial improvement over the initial RTL1000 construct that would allow immediate treatment for acute brain injuries such as TBI and stroke.

### Treatment of acute and chronic EAE with DRa1-MOG-35-55 constructs

**Fig. 1:**
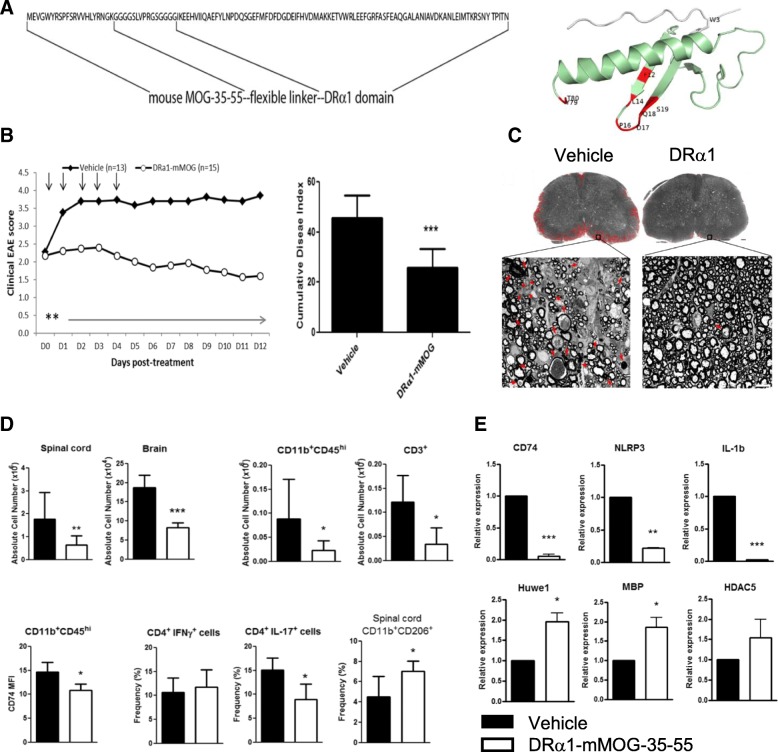
Design and treatment effects of the DRα1-mMOG-35-55 construct in acute EAE: **a** Design, sequence and ribbon model of the DRα1-mMOG-35-55 construct. **b** Treatment of acute EAE in DR*1501-Tg mice with 5 daily doses of vehicle or 100 μg DRα1-mMOG-35-55 starting at a score of ≥ 2 reduced daily (**p < 0.01) and cumulative (***p < 0.001) disease scores and **c** spinal cord histological damage (red) and demyelination as visualized after toluidine blue staining (scale bar 100 mm). High-power view of small areas of lumbar spinal cord (black rectangles) show axons that are undergoing demyelination (red arrow) and cellular infiltration (red arrowhead). Scale bar is 10 mm. **d** DRα1-mMOG-35-55 treatment of C57BL/6 male mice with EAE reduced the absolute numbers of lymphocytes, CD11b^+^CD45^high^ myeloid cells, and CD3^+^ T cells in the spinal cord and brain, expression of CD74 on CD11b^+^CD45^high^ spinal cord cells and intracellular expression of CD4^+^IL-17^+^ T cells in the brain (after stimulation with PMA and ionomycin) and increased the frequency of CD206^+^CD11b^+^CD45^hi^ cells in spinal cords. **e** DRα1-mMOG-35-55 treatment reduced the relative expression of pro-inflammatory genes (CD74, NLRP3, and IL-1b) and increased the expression of genes involved in neurosurvival and regeneration (HUWE1, MBP, and HDAC5) in spinal cord samples from C57BL/6 male mice. *p < 0.05, **p < 0.01, ***p < 0.001. Panels (**a**) (partial), (**b**, **d**, and **e**) reprinted [adapted] from “HLA-DRα1-mMOG-35-55 treatment of Experimental autoimmune encephalomyelitis reduces CNS inflammation, enhances M2 macrophage frequency, and promotes neuroprotection,” by Benedek et al., 2015, Journal of Neuroinflammation, 12:123, Biomedical Central, copyright Benedek et al. 2015 [28]. Reprinted with permission. Published with original ribbon graphic of the DRα1-MOG-35-55 construct. Panel (**c**) reprinted from “HLA-DRα1 constructs block CD74 expression and MIF effects in experimental autoimmune encephalomyelitis,” by Meza-Romero et al., 2014 [22]. Journal of Immunology, 192(9):4164-73, copyright of The American Association of Immunologists. Reprinted with permission

Multiple sclerosis (MS) is a devastating demyelinating neurological disease with an autoimmune origin involving both genetic and environmental factors in disease pathogenesis [23, 24]. Encephalitogenic T cells, macrophages, and microglia play a central role in multiple sclerosis and EAE pathology with T cells and macrophages, migrating across the blood-brain barrier and orchestrating a proinflammatory attack that causes myelin damage and neuronal death leading to the progression of disease severity [23, 25–27]. As discussed above, MIF and D-DT are key cytokines thought to drive the early inflammatory stage of MS to a chronic progressive phase. MIF and DDT levels and have been implicated as markers of clinical worsening in MS and as a requirement for disease progression in EAE. The RTL constructs bind tightly to the MIF/DDT receptor, CD74, and competitively inhibit MIF/DDT binding and downstream signaling through phosphorylated (p)ERK1/2. Due to the ability of our second generation DRα1-MOG-35-55 construct (see sequence and ribbon structure in Fig. 1a) to retain the immunoregulatory activity of the RTL1000 construct, we are now focused on testing DRα1-MOG-35-55 effects on treatment of EAE, with the intent of developing it for use in FDA approved clinical trials in MS and other CNS conditions.

#### DRα1-mMOG-35-55 treatment reverses clinical signs of EAE

As described above, we demonstrated that DRα1-mMOG-35-55 treatment could reverse clinical signs of EAE in DRα1-matched HLA-transgenic DR*1501 mice [22]. We then wished to test if the DRα1-mMOG-35-55 construct could treat EAE in mice that expressed mouse I-A/I-E genes, as opposed to the human HLA-DRα1 and DR2β1 domains expressed in the DR*1501-Tg mice. Indeed, treatment of C57BL/6 mice immunized with mouse (m)MOG-35-55/CFA/Ptx (100 μg daily × 5) after disease onset significantly reduced clinical and histological signs of EAE compared with vehicle-treated mice (Fig. 1b, c) [28]. These results indicate that DRα1-mMOG-35-55 is a potent therapeutic agent to treat EAE in mouse strains expressing different MHC gene sequences but the same disease-inducing mMOG-35-55 peptide.

#### DRα1-mMOG-35-55 treatment reduces the number of CNS-infiltrating cells and their activation state

In order to evaluate effects of DRα1-mMOG-35-55 treatment on migration of activated immune cells into the CNS, mononuclear cells were isolated from brains and spinal cords from C57BL/6 mice 5 days post-EAE treatment. The absolute number of mononuclear cells was reduced both in the brain and spinal cord of DRα1-mMOG-35-55-treated vs. vehicle-treated mice with EAE (Fig. 1d) [28]. This reduction was reflected in the absolute number of infiltrating monocytes and activated resident microglia (CD11b^+^CD45^hi^) as well as CD3^+^ T cells in the spinal cord (*p* < 0.05; Fig. 1d). In addition, the CD74 expression level on CD11b^+^CD45^hi^ cells was significantly lower in the spinal cords of DRα1-mMOG-35-55- vs. vehicle-treated mice (*p* < 0.05; Fig. 1d), in accordance with effects of the parent DR*1501 β1α1-mMOG-35-55 RTL construct in DR*1501-Tg mice [15]. Furthermore, there was a significantly lower frequency of PMA/ionomycin-stimulated IL-17^+^ T cells but not CD4^+^ IFN-γ^+^ Τ cells from DRα1-mMOG-35-55-treated mice compared with stimulated cells from vehicle-treated mice (*p* < 0.05; Fig. 1d). Collectively, these data suggest that DRα1-mMOG-35-55 inhibits migration of activated inflammatory cells from the periphery to the CNS of C57BL/6 mice and that the infiltrating cells in the spinal cords of DRα1-mMOG-35-55-treated mice have reduced expression of CD74 and are less inflammatory compared with spinal cord cells from vehicle-treated mice.

#### DRα1-mMOG-35-55 treatment enhances the frequency of CD11b^+^CD206^+^ anti-inflammatory macrophages in spinal cords of mice with EAE

During EAE, proinflammatory macrophages have been shown to induce CNS inflammation, whereas anti-inflammatory macrophages were shown to be involved in neuroprotection and remyelination [29–31]. We analyzed the frequency of activated CD11b^+^CD206^+^ M2-like macrophages in spinal cords of C57BL/6 mice with EAE 24 h after the last treatment with DRα1-mMOG-35-55 or vehicle. As shown in Fig. 1d, the frequency of M2-like macrophages (CD11b^+^CD206^+^) was significantly increased in DRα1-mMOG-35-55-treated vs. vehicle-treated mice (*p* < < 0.05). In contrast, no difference in CD11b^+^CD206^+^ frequency was observed in the periphery [28].

In order to determine whether DRα1-mMOG-35-55 could directly induce M2-like polarization, we isolated spleen cells from untreated C57BL/6 mice with EAE on day 15 post-immunization and stimulated them with LPS, LPS/MIF, LPS/MIF/DRα1-mMOG-35-55, or DRα1-mMOG-35-55 alone for 24 h at 37 °C. As expected, LPS or LPS/MIF treatment reduced the anti-inflammatory CD11b^+^CD206^+^ frequency by ~ 80% (*p* < 0.001). Treatment with LPS/MIF + DRα1-mMOG-35-55 or DRα1-mMOG-35-55 alone significantly increased the frequency of CD11b^+^CD206^+^ cells compared to LPS or LPS/MIF treatment (*p* < 0.001), but only to ~ 40% of control levels. Similar effects were observed in DR*1501-Tg mice (not shown). Taken together, we conclude that DRα1-mMOG-35-55 treatment mainly inhibits the effects of a pro-inflammatory milieu on M1-like polarization but apparently does not directly induce M2-like polarization [28].

#### DRα1-mMOG-35-55 treatment of EAE reduces the expression of pro-inflammatory genes and increases the expression of genes involved in neurosurvival and regeneration

To evaluate DRα1-mMOG-35-55 effects on CNS inflammation during EAE in a more comprehensive manner, we performed microarray analysis on spinal cords from DRα1-mMOG-35-55- vs. vehicle-treated DR*1501-Tg mice with EAE. EAE was induced with mMOG-35-55/CFA/Ptx, and mice were treated with DRα1-mMOG-35-55 (100 μg daily × 3) or vehicle after disease onset at a clinical score of 2. Twenty-four hours after the last treatment, total RNA was isolated from spinal cords and gene expression profiles from pooled RNA were analyzed using the Mouse Gene 2.0 ST Affymetrix GeneChip system. Relative up- or downregulated genes after treatment with DRα1-mMOG-35-55 plotted against their expression level in spinal cords of vehicle-treated mice revealed 1049 probes that were downregulated by twofold or greater. Out of these probes, 160 genes were shown to be involved in inflammation processes. Conversely, 568 probes were upregulated by twofold or greater in the spinal cord of DRα1-mMOG-35-55-treated vs. vehicle-treated mice. Gene ontology analysis of the genes that were upregulated did not reveal a distinct pathway as was observed for downregulated pro-inflammatory response genes. However, several of the genes that were relatively upregulated by DRα1-mMOG-35-55 treatment were shown to be involved in neuroregeneration, including Prosaposin (PSAP), Myocilin (MYOC), E3 ubiquitin ligase Huwe1, and 3-phosphoinositide-dependent protein kinase 1 (PDPK-1) [32–37]. We validated these microarray results by real-time PCR analysis of spinal cord mRNA from three individual mice in each group (Fig. 1e). Indeed, pro-inflammatory genes such as CD74, Nlrp3, and IL-1b were significantly downregulated in spinal cords of DRα1-mMOG-35-55-treated vs. vehicle-treated mice (*p* < 0.001, *p* < 0.01, and *p* < 0.001, respectively), whereas Huwe1 and MBP were significantly upregulated (*p* < 0.05). Histone deacetylase 5 (HDAC5) was also upregulated, although results did not reach significance [38, 39]. These results indicate the CNS of DRα1-mMOG-35-55-treated mice is not only less inflammatory but also that this treatment could inhibit and potentially reverse ongoing demyelination and neurodegeneration [28].

#### Sex-dependent efficacy of DRα1-mMOG-35-55 in treating chronic EAE

**Fig. 2:**
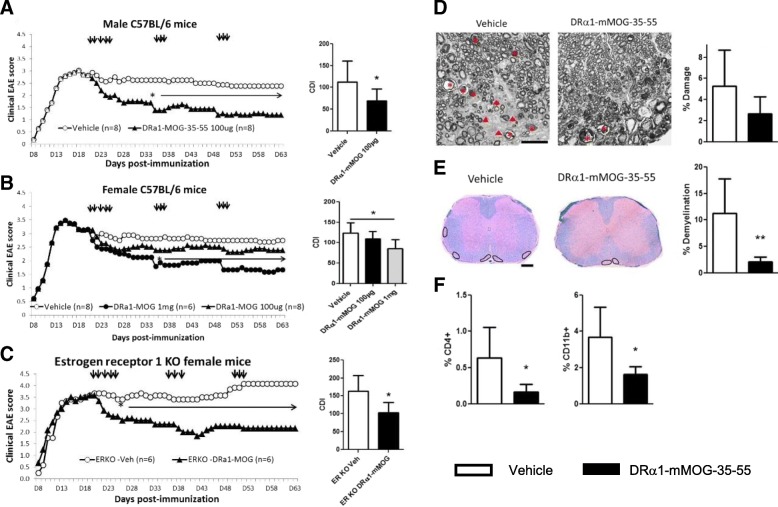
Sex-dependent DRα1-mMOG-35-55 treatment of chronic EAE: **a** DRα1-mMOG-35-55 can treat chronic EAE in C57BL/6 males with repeated 100-μg doses, whereas (**b**) treatment of female C57BL/6 mice with chronic EAE required repeated 1-mg doses except in (**c**) E2 depleted ESR1(ERα)-KO mice that responded to the 100-μg dose. Figures show daily scores (injections indicated by black arrows) and cumulative disease indices. *p < 0.05. **d**, **e** High-dose (1 mg) treatment of females with DRα1-mMOG-35-55 reduced spinal cord damage (trend) and demyelination and (**f**) leukocyte infiltration in female mice with chronic EAE. **d** High magnification (× 63) plastic embedded spinal cord cross-sections are shown from mice 63 days post immunization after toluidine blue staining of lateral spinal cord. Arrowheads show lesion. Asterisks show degenerating axons. Scale bar is 20 μm. **e** Paraffin-embedded sections (× 20) were used for assessing demyelination using LFB-PAS and hematoxylin staining. Scale bar is 200 μm. Percent demyelination is shown in bar graph (right). **p < 0.01. **f** Frequencies of CD4^+^ and CD11b^+^ cells in spinal cord sections are shown for vehicle or 1 mg DRα1-mMOG-35-55-treated female C57BL/6 mice as evaluated by immunofluorescent staining. *p < 0.05. Reprinted from “Sex-dependent treatment of chronic EAE with partial MHC class II constructs,” by Benedek et al., 2017 [40], *J Neuroinflammation, 14(1):100.* Biomedical Central, copyright Benedek et al. 2015 [28]. Reprinted with permission

As shown above, we demonstrated that our second generation pMHC construct, DRα1-mMOG-35-55, could successfully treat EAE when injected into either HLA “matched” DR2-Tg mice or “mismatched” C57BL/6 mice at a clinical score of ≥ 2 [2, 22]. We thus tested the ability of DRα1-mMOG-35-55 to treat chronic EAE in “mismatched” C57BL/6 mice and found an unexpected sex-dependent pattern of treatment, with the 100-μg dose producing a significant improvement of chronic EAE in males (Fig. 2a; *p* < 0.05) but not females (Fig. 2b) [40]. These data were reminiscent of the same sex-dependent response to treatment in experimental stroke (shown below), but in that case, females unresponsive to the 100-μg dose could be effectively treated with a 10-fold higher 1000-μg dose of DRα1-mMOG-35-55 [41]. As demonstrated for the DR*1501-Tg mice, there were no statistical differences in the disease course or the CDI between vehicle-treated male and female C57BL/6 mice. Thus, we repeated the experiment in female C57BL/6 mice with chronic EAE using the 1-mg dose of DRα1-mMOG-35-55 and found a marked early and significant cumulative treatment effect similar to the low-dose treatments in male mice (Fig. 2b; *p* < 0.05). Analysis of spinal cords from DRα1-mMOG-35-55-treated female mice revealed nominally less axonal damage compared to vehicle-treated mice (2.6 ± 0.6% vs. 5.2 ± 1.2%, respectively; Fig. 2d), significantly less demyelination (2.1 ± 0.3% vs. 11.2 ± 2.3%, respectively, *p* < 0.01; Fig. 2e), and reduced inflammation marked by significantly reduced frequencies of CD11b^+^ (1.6 ± 0.1% vs 3.6 ± 0.5%, respectively, *p* < 0.05) and CD4^+^ T cells (0.16 ± 0.04% vs 0.6 ± 0.14%, respectively, *p* < 0.05) (Fig. 2f). These results suggested that the difference in effective dose for treatment of chronic EAE with DRα1-mMOG-35-55 is sex dependent.

#### DRα1-mMOG-35-55 treatment efficacy of female mice depends on estrogen signaling through ERα

Sex hormones have been shown to both positively and negatively regulate the immune system [21, 42, 43]. In order to evaluate the effect of female sex hormones on the treatment response in chronic EAE, WT and ovariectomized (OVX) C57BL/6 female mice were treated with 100 μg DRα1-mMOG-35-55. Unlike the lack of treatment effect in WT C57BL/6 female mice shown above, there was a significant treatment effect with this lower 100-μg dose in OVX females (*p* < 0.05, not shown). To evaluate a specific role for estrogen in regulating the effective dose of RTL constructs, estrogen receptor (ER)α and ERβ knockout (ERKO and BERKO, respectively) mice were treated on day 20 post immunization with 100 μg DRα1-mMOG-35-55, with boosting for 3 days starting on days 35 and 49 post-injection as before. DRα1-mMOG-35-55 treatment significantly reduced disease severity in ERKO (Fig. 2c) but not BERKO (not shown) female mice compared to vehicle-treated mice [40]. Taken together, these data clearly demonstrate that estrogen signaling through ERα affects the potency of partial MHC class II constructs to treat chronic EAE.

### Ischemic injury models of stroke

#### Middle cerebral artery occlusion (MCAO)

Ischemic CNS injury triggering stroke is a leading cause of death and disability worldwide [44, 45], and it is anticipated that nearly 4% of the US population will sustain a stroke by 2030 [46]. Inflammation involving rapid activation of microglia and time-dependent infiltration of activated peripheral immune cells, including neutrophils, macrophages, T cells, and B cells, into the affected brain tissue is a major contributor to onset and progression of stroke [47–52]. In MCAO, the initial influx of inflammatory cells is followed by a profound degree of immunosuppression marked by atrophy of the spleen and thymus [50, 53–55]. While pro-inflammatory immunocytes, including MOG-35-55-specific T cells [56], exacerbate stroke, alternatively activated macrophages and microglia provide a critical balance for regulating inflammation and tissue repair [31, 57].

Currently, tissue plasminogen activator (tPA) remains the only FDA-approved drug for ischemic stroke, and there is a crucial unmet need for new drugs that meet the Stroke Academic Industry Roundtable (STAIR) criteria. According to these criteria, an optimal therapy should exhibit the following characteristics: (1) efficacy in aged animals and animals with co-morbidity, (2) efficacy in both male and female animals, (3) compatible interaction with tPA, (4) biomarker endpoints (MRI, serum markers), and (5) reproducibility in at least one independent laboratory [58]. As reviewed previously, our RTL constructs have been shown to reduce infarct volume and neurological deficit in various cerebral ischemia models in young adult and aging male and female mice. In addition, partial MHC class II constructs were shown to reverse stroke-associated splenic atrophy and promote a protective anti-inflammatory macrophage/microglia phenotype in the CNS which contributes to tissue repair and recovery after stroke [59, 60]. A recent study has also demonstrated increased expression of CD74 on T cells and antigen-presenting cells that directly correlate with infarct size and neurological deficits in people with ischemic stroke [61].

##### DRα1-mMOG-35-55 treatment significantly reduces infarct size after MCAO in DR2-Tg mice

**Fig. 3:**
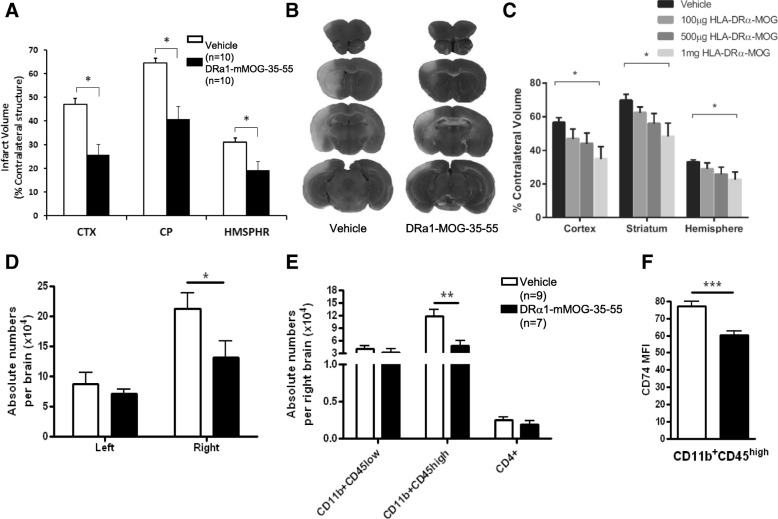
DRα1-mMOG-35-55 treatment of transient MCAO: **a** Four daily 100 μg DRα1-mMOG-35-55 treatments significantly decreased infarct volumes in DR*1501-Tg male mice after 60 min MCAO and 96 h of reperfusion in cortex (CTX), caudateputamen (CP), and total hemisphere (HMSPHR) (*p ≤ 0.05). **b** Representative 2,3,5-triphenyltetrazolium chloride stained cerebral sections after 96 h of reperfusion are shown following 1 h of MCAO. **c** Treatment of female DR*1501-Tg mice required four daily injections of 1 mg DRα1-mMOG-35-55 to significantly reduce infarct volumes (*p ≤ 0.05). **d** DRα1-MOG-35-55 treatment reduced the absolute number of infiltrating cells, **e** activated CD11b^+^CD45^high^ cells, and **f** their CD74 surface expression in the ischemic brain in male mice 96 h after MCAO. *p < 0.05, **p < 0.01, ***p < 0.001. Panels (**a**, **b**, **d**, **e**) reprinted by permission from RightsLink: Springer, Metabolic Brain Disease, A novel HLA-DRα1-MOG-35-55 construct treats experimental stroke, Benedek et al., copyright 2014 [72]. Panel (**c**) reprinted from “Novel humanized recombinant T cell receptor ligands protect the female brain after experimental stroke,” by Pan et al., 2014, Translational Stroke Research, 5(5):577-85, copyright Pan et al., 2014 [63]

Evaluation of brain infarcts 96 h after MCAO demonstrated that DRα1-mMOG-35-55-treated male DR2-Tg mice had significantly reduced infarct volumes compared with the vehicle-treated group as shown in Fig. 3a [62]. Cortical infarct volume was 25.9 ± 4.67% in DRα1-mMOG35-55-treated compared to 47.0 ± 2.5% in vehicle-treated mice (*p* < 0.01). Similarly, striatal infarct volume was 40.8 ± 5.4% vs. 64.5 ± 2.0% respectively in DRα1-mMOG-35-55-treated vs. vehicle-treated mice (*p* < 0.01), whereas the total hemispheric infarct volume was 19.4 ± 3.6% vs. 31.1 ± 1.71%, respectively (*p* < 0.01). Quantitative assay of TTC stained cerebral sections after 96 h of reperfusion illustrated the smaller infarct area in DRα1-mMOG-35-55-treated mice compared with vehicle-treated mice (Fig. 3b). There were no significant differences in laser-Doppler perfusion before, during, or immediately after MCAO between DRα1-MOG-35-55-reated and vehicle-treated groups.

##### DRα1-mMOG-35-55 reduces infarct volumes in a dose-dependent manner in female mice

Similar to the requirement for higher drug doses for treating female mice during chronic EAE shown above, treatment with 1 mg DRα1-mMOG-35-55 in DR*1501 female mice also significantly reduced infarct volume relative to vehicle treatment, as shown in Fig. 3c [63]. Cortical volume was 34.9 ± 7.3% in DRα1-mMOG-35-55-treated vs. 56.5 ± 2.9% in vehicle-treated mice (*p* = 0.007); for striatum, volumes were 48.2 ± 8.0% vs. 69.7 ± 3.7%, respectively (*p* = 0.02); and for hemisphere, volumes were 22.8 ± 4.4% vs. 33.1 ± 1.3%, respectively (*p* = 0.02). In contrast to treatments with two domain RTL1000, 100-μg and 500-μg doses of DRα1-mMOG-35-55 did not significantly improve infarct volumes relative to the vehicle-treated group. No differences were observed in intra-ischemic LDF or temporalis muscle temperature among the various groups, nor were there any differences in body weight, health scores, or mortality rates.

##### DRα1-mMOG-35-55 reduces the number of activated microglia and infiltrating monocytes and their CD74 cell surface expression in the ischemic brain

We sought to determine if the reduced infarct size after DRα1-MOG-35-55 treatment was due to diminished number of infiltrating inflammatory cells in the brain after MCAO. DRα1-mMOG-35-55 treatment significantly reduced the absolute number of mononuclear cells in the right ischemic brain compared with vehicle-treated mice (*p* < 0.05) (Fig. 3d) [62]. This difference is attributed mainly to the reduction in the number of activated CD11b^+^CD45^high^ monocytes (*p* < 0.01; Fig. 3e). There were no significant differences in the absolute numbers of CD4^+^ T cells or resting microglia (CD11b^+^CD45^low^). In the non-ischemic left brain, there were no differences in the total absolute number of mononuclear cells or in any specific cell type.

As shown above, we demonstrated that DRα1-mMOG-35-55 treatment of EAE led to a reduction of activated CD11b^+^ cells in the spinal cord and that DRα1-mMOG-35-55 reduced the cell surface expression of the MIF receptor, CD74, on the activated CD11b^+^ cells. In order to determine if DRα1-mMOG-35-55 treatment had the same effect in MCAO, expression of CD74 cell surface levels was evaluated on CD11b^+^CD45^high^ cells in the right ischemic hemisphere. As shown in Fig. 3f, there was a significant reduction in the level of CD74 MFI expression in the DRα1-mMOG-35-55-treated mice compared with the vehicle-treated mice (*p* < 0.01). Thus, as in EAE, DRα1-mMOG-35-55 can reduce the number of activated microglia and infiltrating monocytes in the ischemic brain and reduce the expression of CD74 on the surface of these cells.

##### DRα1-mMOG-35-55 treatment affects the immune gene expression profile in the ischemic brain after MCAO

In order to assess the DRα1-mMOG-35-55 treatment effect on the expression of immune-related genes in brain, mRNA was isolated from the ischemic brains of 3 vehicle-treated mice and 3 DRα1-mMOG-35-55-treated mice. A real-time PCR assay was performed on pooled cDNA samples, and expression levels of the DRα1-mMOG-35-55-treated sample were analyzed relative to the vehicle-treated sample. Validation of 3 genes, IL-4, CCL3, and ACE using individual samples, demonstrated that the expression trends were the same as in the immune array (not shown). The immune array data demonstrated that there was a decrease in the expression of monocyte-related genes such as CCL3 and CCL2 and increase in Th1 and Th2-related genes such as IL-12, Tbx21, IL-4, and IL-13 [62]. It is important to note that several genes that were associated previously with cerebrovascular function and ischemic brain injury, including ACE and EDN1, were downregulated after DRα1-mMOG-35-55 treatment compared to vehicle treatment.

### Permanent distal middle cerebral artery occlusion (dMCAO)

Post-stroke inflammation includes a rapid activation of microglia followed by the infiltration of peripheral inflammatory cells, including neutrophils, T cells, B cells, and macrophages [64–67]. The influx of these immune cells is known to exacerbate brain injury and deteriorate stroke outcomes [68, 69]. Therefore, reducing the brain infiltration of peripheral immune cells and reducing cerebral inflammation after stroke may represent effective therapeutic strategies [70]. Since many cases of stroke in humans are non-reperfused, we tested the effect of DRα1-MOG-35-55 in a permanent distal MCAO model of stroke [71].

#### DRα1-mMOG-35-55 treatment significantly reduces infarct size at 4 days after dMCAO

**Fig. 4:**
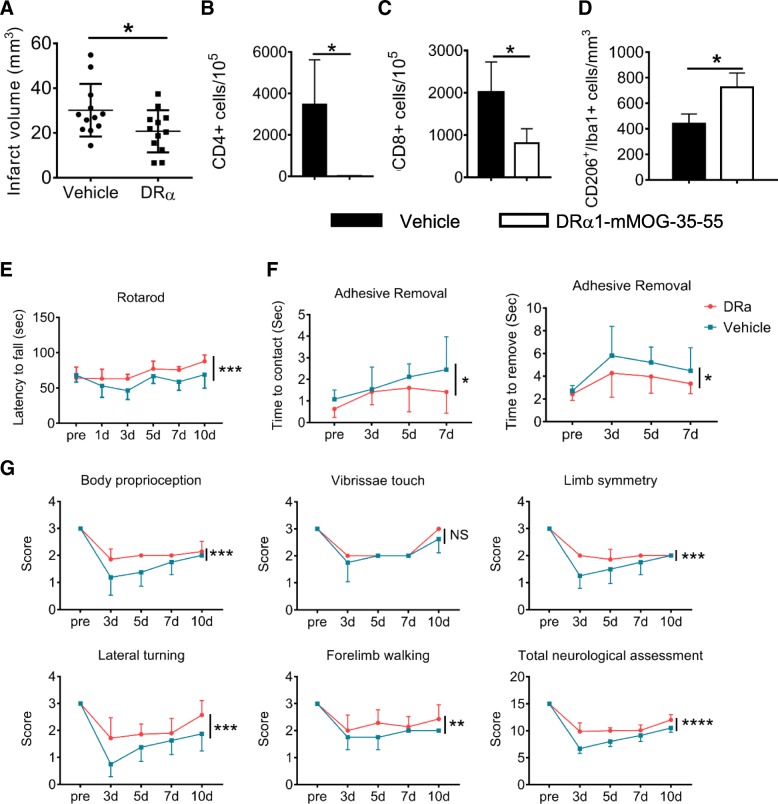
DRα1-mMOG-35-55 treatment of permanent MCAO: **a** Four daily 100 μg DRα1-mMOG-35-55 treatments significantly reduced infarct volumes and **b** infiltration of CD4^+^ and **c** CD8^+^ T cells and **d** increased numbers of CD206^+^Iba1^+^ microglia/macrophages into the ischemic hemisphere of C57BL/6 male mice 96 h after induction of dMCAO (*p < 0.05). Mice treated with DRα1-mMOG-35-55 or vehicle were evaluated for sensorimotor functions 10 days after dMCAO and demonstrated significant improvement in (**e**) Rotarod test, (**f**) Adhesive removal test and (**g**) Modified Garcia scores (total neurologic assessment) that included body proprioception, vibrissae touch (NS), limb symmetry, lateral turning, and forelimb walking. NS no significance. *p < 0.05; **p < 0.01; ***p < 0.001; ****p < 0.0001. Reprinted by permission from RightsLink: Springer, Translational Stroke Research, DRα1-MOG-35-55 Reduces Permanent Ischemic Brain Injury, Wang et al., copyright 2017

Daily subcutaneous treatment with DRα1-mMOG-35-55 for four consecutive days significantly reduced the infarct volume 4 days after the onset of dMCAO as compared with vehicle-treated mice (Fig. 4a; *p* < 0.05).

#### DRα1-MOG-35-55 treatment inhibits influx of T lymphocytes into brain after dMCAO

We then evaluated the effects of DRα1-mMOG-35-55 treatment on immune cell infiltration into the ischemic brain 4 days after dMCAO. The brain tissue was collected from the ipsilateral brain, and single-cell suspension was prepared for FACS analysis. The results showed that DRα1-mMOG-35-55 treatment markedly decreased the number of CD3^+^CD4^+^ T cells (Fig. 4b) and CD3^+^CD8^+^ T cells (Fig. 4c) in the ischemic hemisphere compared to those of the vehicle-treated mice.

#### DRα1-mMOG-35-55 treatment enhances polarization of anti-inflammatory microglia/macrophages in the ischemic brain after dMCAO

It has been demonstrated that DRα1-mMOG-35-55 treatment can reduce the number of activated macrophages in the transient MCAO model [72]. Therefore, we sought to assess whether DRα1-mMOG-35-55 treatment could also reduce the number of activated microglia/macrophage in the distal permanent model of stroke. CD11b^+^CD45^int^ microglia and CD11b^+^CD45^high^ macrophages were distinguished by flow cytometry. The total number of macrophages and microglia showed no difference between DRα1-mMOG-35-55-treated and vehicle-treated mice at day 4 after dMCAO. Immunofluorescent staining of F4/80 confirmed comparable numbers of F4/80+ macrophages in the ischemic areas in DRα1-mMOG-35-55-treated and vehicle-treated mice. Interestingly, the expression of the M2-like marker, CD206, was significantly upregulated in Iba1^+^ microglia/macrophage in DRα1-mMOG-35-55-treated mice as compared to vehicle-treated controls (Fig. 4d). These results suggest that although DRα1-mMOG-35-55 does not reduce the number of activated microglia/macrophage after dMCAO, it can shift microglia/macrophage toward a beneficial anti-inflammatory phenotype.

#### DRα1-mMOG-35-55 treatment reduces the gene expression of inflammatory cytokines in the ischemic brain after distal MCAO

In order to evaluate the effect of DRα1-mMOG-35-55 treatment on cerebral inflammation after dMCAO, we measured the mRNA expression of several pro-inflammatory cytokines in the ischemic brain using RT-PCR at 4 days after dMCAO. DRα1-mMOG-35-55 treatment significantly reduced the expression of IL-1α and IL-17 in ipsilateral hemispheres compared to that of vehicle-treated mice, although the expression of TNF-α or IFN-γ was not statistically different.

### Methamphetamine addiction

Methamphetamine use causes long-term neuropsychiatric impairments that make addiction to this drug extremely challenging to treat [73, 74]. Relapse rates following current methamphetamine abuse treatments are very high (~ 40–60%) [75], and the neuropsychiatric impairments (e.g., cognitive deficits, mood disorders) that arise and persist during remission from methamphetamine addiction likely contribute to these high relapse rates [76]. Pharmacotherapeutic development of medications to treat addiction has focused on neurotransmitter systems with only limited success, and there are no Food and Drug Administration-approved pharmacotherapies for methamphetamine addiction. Methamphetamine dependence is associated with long-term structural damage to regions of the brain that control cognitive and behavioral function [77–79]. A growing literature shows that methamphetamine alters peripheral and central immune functions and immune factors such as cytokines, chemokines, and adhesion molecules play a role in the development and persistence of methamphetamine-induced neuronal injury and neuropsychiatric impairments [80, 81].

These observations that parallel immune-mediated damage in other conditions marked by CNS damage discussed in this review provided a strong rationale for use of RTL constructs for treatment of methamphetamine addiction. We thus evaluated the efficacy of the RTL551 construct (I-A^b^ β1α1 domains linked to mMOG-35-55), in treating learning and memory impairments induced by repeated methamphetamine exposure. C57BL/6 mice were exposed to two different methamphetamine treatment regimens (using repeated doses of 4 mg/kg or 10 mg/kg, s.c.). Cognitive performance was assessed using the Morris water maze and CNS cytokine levels were measured by multiplex assay. Immunotherapy with RTL551 improved the memory impairments induced by repeated methamphetamine exposure in both mouse models of chronic methamphetamine addition. Treatment with RTL551 also attenuated the methamphetamine-induced increases in hypothalamic interleukin-2 (IL-2) levels. Collectively, these initial results indicate that neuroimmune targeted therapies, potentially including DRα1-hMOG-35-55 constructs, may have potential as treatments for methamphetamine-induced neuropsychiatric impairments [82].

### Traumatic brain injury

Traumatic brain injury (TBI) results in severe neurological impairments without effective treatments. The primary injury occurs upon impact, disrupting the blood-brain barrier and blood vessels that cause brain edema [83]. Inflammation involving migration of activated peripheral immune cells and resident microglia and astrocytes appears to be an important contributor to key pathogenic events that result in secondary brain injury following TBI [83–86] and therefore serves as a promising target for novel anti-inflammatory therapies. As shown above, we have demonstrated the ability of the DRα1-mMOG-35-55 construct to reduce CNS inflammation and tissue injury in animal models of multiple sclerosis, ischemic injury, and methamphetamine addiction. Thus, we sought to determine if DRα1-mMOG-35-55 treatment of a fluid percussion injury (FPI) mouse model of TBI could reduce the lesion size and improve disease outcome measures [87].

#### DRα1-mMOG-35-55 attenuates neurodeficits and lesion volume in FPI mice

**Fig. 5:**
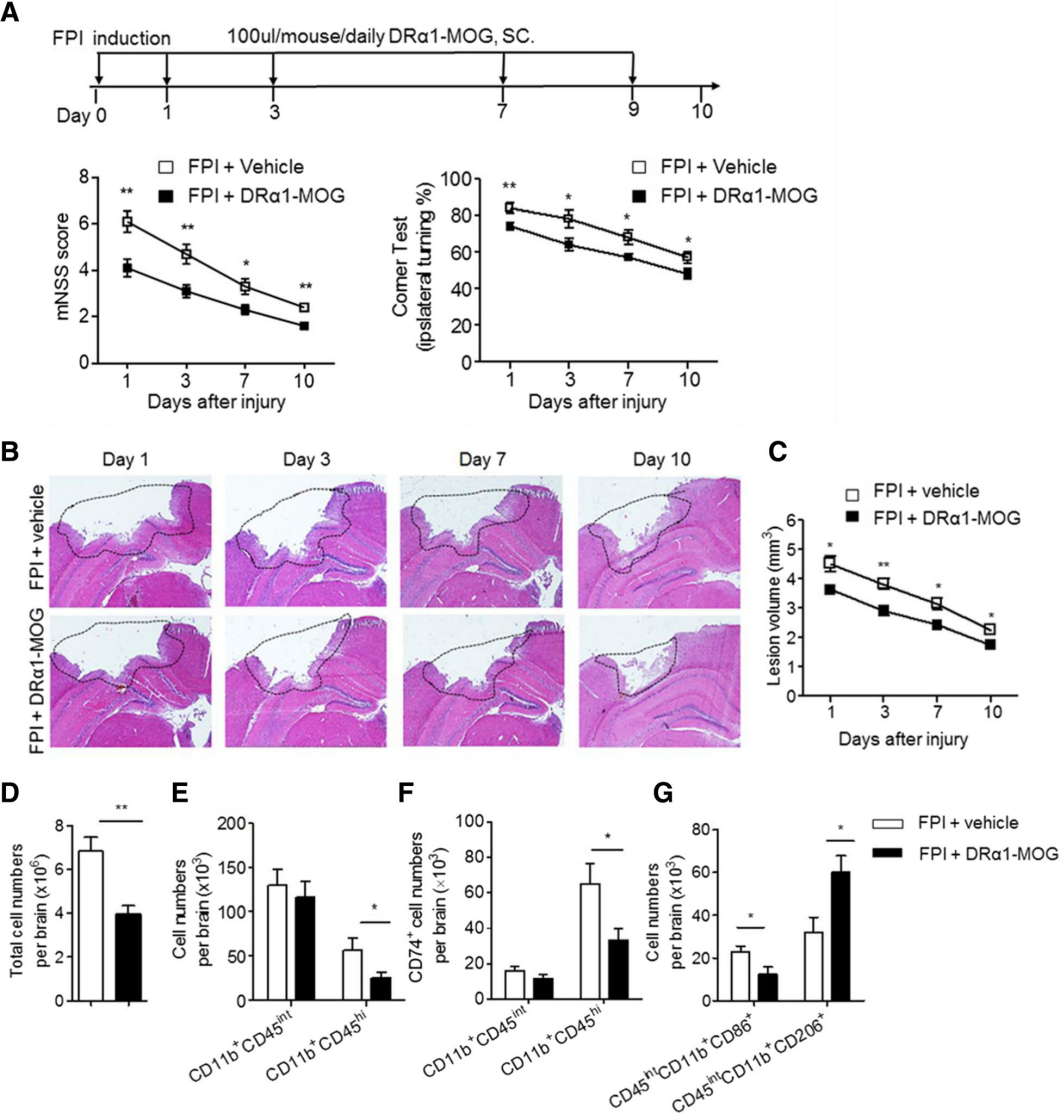
DRα1-mMOG-35-55 treatment of TBI: **a** DRα1-MOG-35-55 treatment (100 μg in 0.1 ml. s.c. given over 10 days starting immediately after FPI) of C57BL/6 male mice reduced TBI neurodeficits, including the modified Neurological Severity Score (mNSS) to evaluate motor, sensory, and balance functions and the corner turning test. *p < 0.05, **p < 0.01. DRα1-mMOG-35-55 treatment reduced lesion size as evaluated by **b** H&E images of coronal brain tissue sections **c** quantified at indicated time points after TBI, and **d** reduced total cell numbers and **e** activated CD11b^+^CD45^hi^ microglia/monocytes in the brain and **f** their surface expression of CD74. **g** Conversely, DRα1-mMOG-35-55 treatment reduced numbers of CD45^int^CD11b^+^CD86^+^ and increased numbers of CD45^int^CD11b^+^CD206^+^ microglia on day 3 after TBI. *p < 0.05, **p < 0.01. Reprinted by permission from RightsLink: Springer, Translational Stroke Research, DRα1-MOG-35-55 treatment reduces lesion volumes and improves neurological deficits after traumatic brain injury, Yang et al., copyright 2017 [61]

To assess the therapeutic effects of DRα1-mMOG-35-55 after TBI, we measured neurological impairment scores and lesion volumes in mice subjected to FPI. Mice received nine daily s.c. injections of 100 μg DRα1-mMOG-35-55 or vehicle starting immediately after FPI (Fig. 5a) and were assessed intermittently and 24 h after the final treatment (day 10) using the modified Neurological Severity Score (mNSS) and the Corner Turning test. DRα1-mMOG-35-55 treatment significantly reduced both neurodeficit measures (Fig. 5a) as well as lesion volumes (Fig. 5b) at all time points measured after TBI compared to vehicle controls (Fig. 5c).

#### DRα1-mMOG-35-55 reduces brain infiltration of CD11b^+^ cells and their expression of CD74

To determine whether DRα1-mMOG-35-55 treatment of brain injury could affect the cell numbers and CD74 expression of CD11b^+^ cells in the brain, we assessed numbers of non-activated brain-intrinsic microglia (CD11b^+^CD45^int^) and brain-infiltrating macrophages/activated microglia (CD11b^+^CD45^hi^) after TBI. We found that DRα1-mMOG-35-55 treatment significantly reduced the total number of brain-infiltrating cells (*p* = 0.01) (Fig. 5d), with predominant effects on CD11b^+^CD45^hi^ macrophages/activated microglia (Fig. 5e) that expressed CD74 (*p* = 0.03) (Fig. 5f). In contrast, no changes were observed in total numbers or CD74 expression of non-activated CD11b^+^CD45^int^ microglia after treatment with DRα1-mMOG-35-55 (*p* = 0.12). These results thus demonstrate that treatment with the DRα1-mMOG-35-55 construct can reduce the infiltration and local activation of CD11b^+^CD45^hi^ cells in the injured brain and their expression of CD74 after TBI. CD86 and CD206 are two markers routinely used to respectively characterize the pro-inflammatory versus anti-inflammatory phenotypes of CD11b^+^CD45^int^ cells. We therefore determined the expression of CD86 and CD206 by brain-infiltrating CD11b^+^CD45^int^ cells. The results clearly demonstrated a reduction of CD86-expressing inflammatory CD11b^+^CD45^int^ cells (*p* = 0.03) and an increase of anti-inflammatory CD206-expressing CD11b^+^CD45^int^ cells after treatment with DRα1-mMOG-35-55 (*p* = 0.02) (Fig. 5g).

This study provides the first evidence that DRα1-MOG-35-55 treatment improves the outcome of TBI. Our results demonstrate that daily s.c. administration of DRα1-MOG-35-55 can significantly enhance functional outcomes, reduce brain lesion size, block infiltration of CD11b^+^ cells into the injured brain, and promote an anti-inflammatory phenotype in activated CD11b^+^CD45^hi^ cells that infiltrate from the periphery or that result from local activation of resident CD11b^+^CD45^int^ microglia after TBI [87]. These anti-inflammatory changes observed in the injured brain were in stark contrast with increased numbers of activated monocytes in the blood that were inhibited from crossing the blood-brain barrier into the CNS and the lack of any demonstrable changes in the spleen (not shown).

### DRhQ as the next generation anti-inflammatory drug for CNS pathological conditions

An analysis of the alignment of several human Class II α1 domains revealed a unique characteristic of the DRα1 domain not shared with other human or mouse class II [88]. This unique feature shows a glutamine (Q) residue at position 18 in most of the sequences used in the alignment. The DRα1 domain stands apart from the other sequences, however, with a leucine (L) at position 18. It is noteworthy that along with the other peptide-presenting Class II domains, this Q18 residue is also conserved among the non-antigen presenting proteins DMα1 and DOα1. In all the analyzed Class II α1 domain primary sequences and the crystal structures stored in the Protein Data Blank (PDB), this region localizes in the loop between β-strand 1 and β-strand 2 at the N-terminus of the polypeptide pointing outwards of the bulk of the molecule. Based on this information, we introduced an amino acid substitution of glutamine for leucine at position 18 of the DRα1 domain (position 50 of the DRα1-hMOG-35-55 construct) termed DRhQ that was found to enhance the binding affinity of the construct for CD74 by 8–10-fold. This substitution did not affect the structure of the molecule as evaluated by circular dichroism and conformational antibody probing. With regard to potential clinical application, the increased binding affinity translated into a commensurate ability of DRhQ to competitively inhibit MIF binding to its cognate CD74 receptor and significantly enhanced the ability of the construct to treat ongoing clinical signs of severe EAE. Moreover, DRhQ treatment of WT C57BL/6 mice with EAE reduced the increased pERK1/2 phosphorylation levels in vitro in splenocytes to a background level. These data suggest that binding affinity for CD74 could serve as an in vitro indicator of DRhQ potency and reduced pERK levels in peripheral blood mononuclear cells after injection, an in vivo indicator of DRhQ biological activity. These measures of activity will be of value when seeking FDA approval for use of the DRhQ construct as an Investigational New Drug in human clinical trials.

## Summary and conclusions

**Fig. 6:**
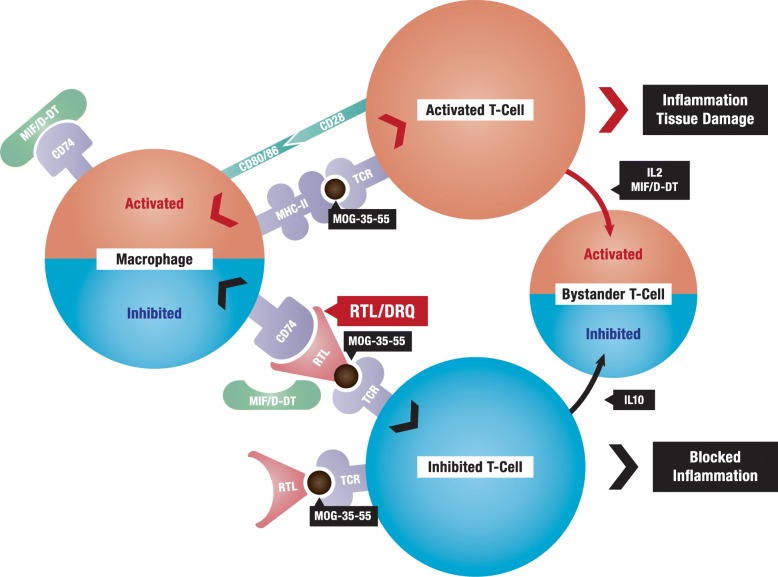
Bifunctional immunosuppressive mechanism of RTL, DRα1-MOG-35-55, and DRQ. Graphic shows dual interactions of partial MHC constructs that simultaneously reduce TCR signaling to enhance IL-10 secretion by MOG-35-55-specific T cells and competitively inhibit MIF and D-DT binding and signaling through CD74 on macrophages and dendritic cells. Original figure

DRα1-mMOG-35-55 treatment of EAE, ischemic injury, methamphetamine addiction and TBI results in highly significant clinical and histological improvement as well as increased neurological functionality. This is likely due to the striking ability of DRα1-mMOG-35-55 and DRhQ to modulate common inflammatory pathways (illustrated in Fig. 6) and enhance remyelination and neuroprotection through dual regulatory mechanisms. This review provides evidence from several CNS inflammatory conditions showing that the DRα1 construct can strongly inhibit the activation and recruitment of brain-infiltrating T cells and CD11b^+^CD45^hi^ myeloid cells that have increased expression of CD74 subsequent to CNS damage. DRα1-mMOG-35-55 also reduced expression of the co-stimulatory CD86 marker on CD11b^+^CD45^hi^ cells but enhanced expression of the anti-inflammatory CD206 marker on CD11b^+^CD45^int^ microglial cells within the CNS. Establishing the common therapeutic activity of DRα1-mMOG-35-55 for these various CNS conditions in mouse models portends well for successful treatment of these and other CNS conditions by our more potent next generation DRhQ construct designed for human use.

## Abbreviations

(h)MOG-35-55: Human
(m)MOG-35-55: Mouse
(p)ERK1/2: Phosphorylated
APC: Antigen presenting cells
CNS: Central nervous system
D-DT=MIF-2: d-Dopachrome tautomerase
dMCA: Permanent distal middle cerebral artery occlusion
EAE: Experimental autoimmune encephalomyelitis
ERK: Extracellular-regulated kinase
ERα or ERKO: Estrogen receptor alpha
ERβ or BERKO: Estrogen receptor beta
FACS: Fluorescence-activated cell sorting
FPI: Fluid percussion injury
GFP: Green fluorescent protein
HDAC5: Histone deacetylase 5
IL-2: Interleukin-2
L: Leucine
MBP: Myelin basic protein
MCAO: Middle cerebral artery occlusion
MIF: Macrophage migration inhibitory factor
mNSS: Modified neurological severity score
MOG: Myelin oligodendrocyte glycoprotein
MS: Multiple sclerosis
MYOC: Myocilin
OVX: Ovariectomized
PDB: Protein Data Blank
PDPK-1: 3-Phosphoinositide-dependent protein kinase 1
PLP: Proteolipid protein
pMHC: Partial major histocompatibility complex class II constructs
PSAP: PROSAPOSIN
Q: Glutamine
RTL: Recombinant T cell receptor ligand
SAR: Structure activity relationship
STAIR: Stroke Academic Industry Roundtable
TBI: Traumatic brain injury
TCR: T cell receptor
tPA: Tissue plasminogen activator
WT: Wild-type
